# MRI following scoliosis surgery? An analysis of implant heating, displacement, torque, and susceptibility artifacts

**DOI:** 10.1007/s00330-020-07546-6

**Published:** 2020-12-04

**Authors:** Andreas Heinrich, Maximilian Reinhold, Felix V. Güttler, Georg Matziolis, Ulf K.-M. Teichgräber, Timo Zippelius, Patrick Strube

**Affiliations:** 1 Department of Radiology, Jena University Hospital – Friedrich Schiller University, Am Klinikum 1, 07747 Jena, Germany; 2 Department of Orthopedics, Jena University Hospital, Campus Eisenberg, Klosterlausnitzer Straße 81, 07607 Eisenberg, Germany

**Keywords:** Cobalt, Magnetic resonance imaging, Safety, Spine, Titanium

## Abstract

**Objectives:**

The implant constructs used in scoliosis surgery are often long with a high screw density. Therefore, it is generally believed that magnetic resonance imaging (MRI) should not be carried out after scoliosis surgery, with the result that computed tomography is often preferred despite the ionizing radiation involved. The objective of this study was to evaluate the MRI compatibility of long pedicle-screw-rod constructs at 1.5 T and 3 T using standardized methods of the American Society for Testing and Materials (ASTM).

**Methods:**

Constructs between 130 and 430 mm long were systematically examined according to the ASTM standards F2182 (radio frequency–induced heating), F2119 (susceptibility artifacts), F2213 (magnetically induced torque), and F2052 (magnetically induced displacement force).

**Results:**

The maximum heating in the magnetic field was 1.3 K. Heating was significantly influenced by magnetic field strength (*p* < 0.001), implant length (*p* = 0.048), and presence of cross-links (*p* = 0.001). The maximum artifact width for different lengths of the anatomically bent titanium rods with CoCr alloy ranged between 14.77 ± 2.93 mm (TSE) and 17.49 ± 1.82 mm (GRE) for 1.5 T and between 23.67 ± 2.39 mm (TSE) and 27.77 ± 2.37 mm (GRE) for 3 T. TiCP and TiAl showed the smallest and CoCr and CoCr Plus the largest artifact widths. The magnetically induced torque and displacement force were negligible.

**Conclusions:**

MRI following scoliosis surgery with long implant constructs is safe with the patient in supine position. Although susceptibility artifacts can severely limit the diagnostic value, the examination of other regions is possible.

**Key Points:**

• *Large spinal implants are not necessarily a contraindication for MRI; MR conditional status can be examined according to the ASTM standards F2182, F2119, F2213, and F2052.*

• *A metallic pedicle-screw-rod system could be reliably and safely examined in all combinations of length (130 to 430 mm), configuration, and material in a B*_*0*_ *at 1.5 T and 3 T.*

• *According to ASTM F2503, the examined pedicle-screw-rod system is MR conditional and especially the young patients can benefit from a non-ionizing radiation MRI examination*.

## Introduction

The use of metallic implants such as pedicle screws and rods is currently standard in spinal surgery to treat deformity, degeneration, destruction, and trauma [1, 2]. Especially in patients with deformity, the implants may be long and the constructs may have a high density of screws. The compatibility of various orthopedic implants with magnetic resonance imaging (MRI) has been studied [2–4], but the modern metallic pedicle-screw-rod systems have not been sufficiently investigated in this regard. A previously published study [1] on a pedicle-screw-rod system focused mainly on shorter implant constructs and used a field strength of 7 T. However, the pedicle screw density used here was lower and the 7-T magnetic field strength was higher than in routine clinical care because usually the patients are examined at 1.5 T and 3 T. Furthermore, not all aspects of MRI compatibility were examined; no attention was paid, for example, to magnetically induced torque or susceptibility artifacts. Patients with long metallic pedicle-screw-rod constructs who need a tomographic examination, e.g., in the presence of postoperative complications or in scoliosis secondary to tumors such as neurofibromas, often undergo computed tomography (CT) despite the exposure to ionizing radiation. Particularly due to the youth of many scoliosis patients, MRI would be preferable.

Before an examination with a given MRI scanner, it must be ensured that any metallic implants in the patient are MR conditional, i.e., pose no known hazards in a specified MRI environment with specified conditions of use. The American Society for Testing and Materials (ASTM) has defined standard methods for evaluation of passive implants to determine whether they are MR conditional and safe. During MRI, radiofrequencies (RF) are emitted with high energy, which can lead to induced heating of, and/or in the vicinity of, a passive medical implant (ASTM F2182 [5]). This heating can lead to injuries [6–8] and is one of the most common MRI incidents. Furthermore, the diagnostic value of MRI can be reduced by susceptibility artifacts [9, 10]. These artifacts are characterized by distorted object geometry and artificial signal variations in the MR image caused by large susceptibility gradients between an implant and neighboring tissues (ASTM F2119 [11]). Additionally, even in the homogeneous area of the static magnetic field (B_0_), strong magnetically induced torques may act on implanted ferromagnetic medical devices (ASTM F2213 [12]). These forces are liable to restrict the function of the device, damage it, and/or cause severe, sometimes life-threatening, incidents [13–15]. However, most serious injuries are caused by the magnetically induced displacement force [8, 16], because the spatial gradient of B_0_ can greatly accelerate ferromagnetic objects or metallic implants (ASTM F2052 [17]).

The objective of this study was to evaluate the MRI safety and MR conditional status of a metallic pedicle-screw-rod system of different lengths (130 to 430 mm), configurations, and materials at field strengths of 1.5 T and 3 T adapted to a full-spine sawbone profile based on standardized testing methods of the ASTM.

## Materials and methods

**Fig. 1:**
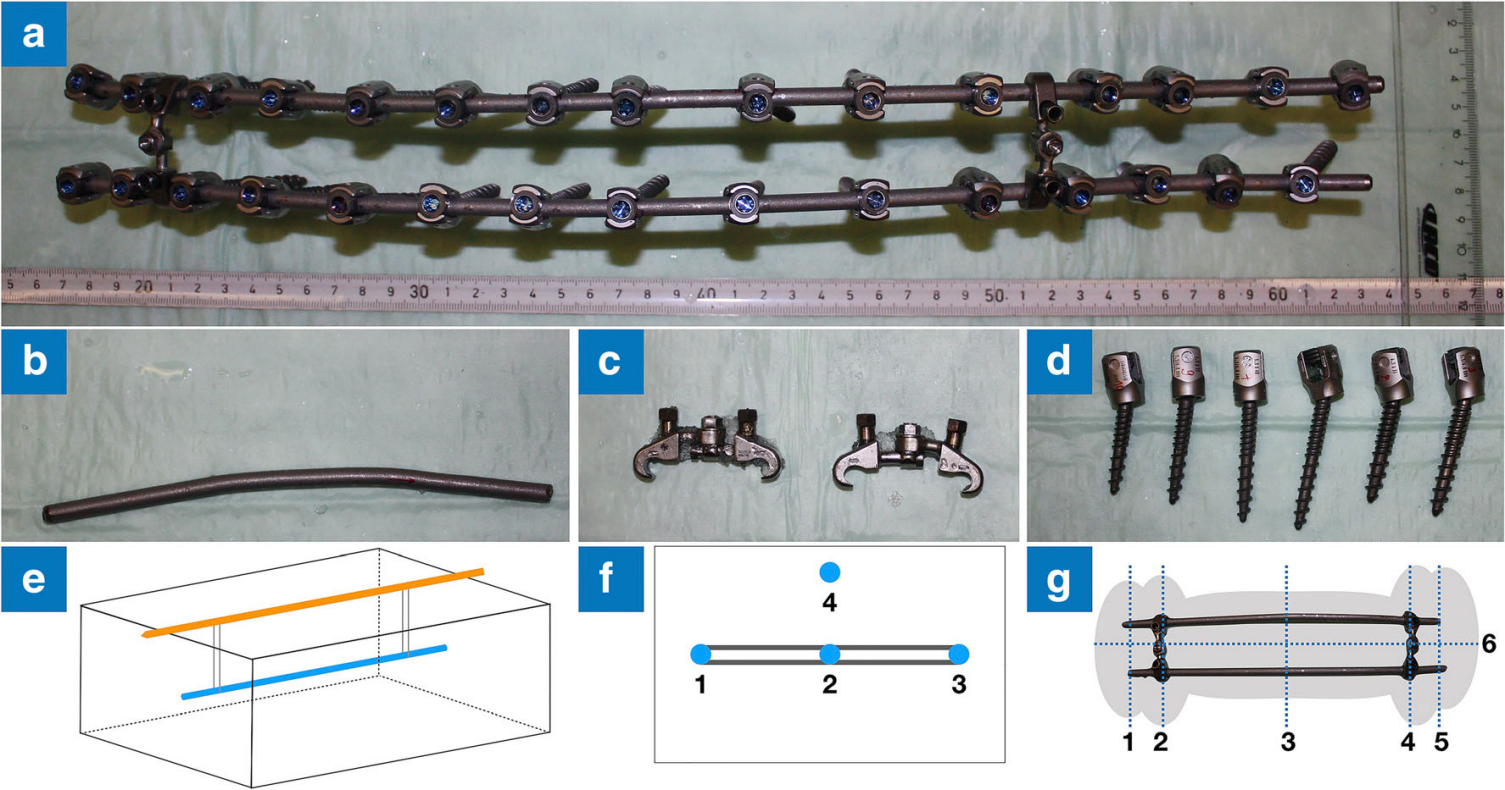
The implant examined in this study with (**a**) a pedicle-screw-rod system consisting of (**b**) titanium with cobalt-chromium alloy rods, (**c**) titanium cross-links, and (**d**) titanium with cobalt-chromium alloy screws. For the measurements, (**e**) the implant was hung from a wooden rod using thin plastic cords. The RF-induced heating was measured (**f**) at four points (1–3: middle and ends of implant; 4: reference). The susceptibility artifact was measured (**g**) at six points (1, 3, and 5: middle and ends of implant; 2 and 4: at position of cross-link; 6: along longitudinal axis of implant)

In the present study, a pedicle-screw-rod system (CD HORIZON® SOLERA^TM^ Spinal System, Medtronic) was mounted, the rods were bent to the anatomical shape, and screw length, position, and dimensions were adapted to the anatomical dimensions of a full-spine sawbone from thoracic vertebra 3 to lumbar vertebra 5 (14 segments, 430 mm, Fig. 1a–d). This implant construct was systematically evaluated with standardized methods of the ASTM at field strengths of 1.5 T (Magnetom Avanto and Aera, Siemens) and 3 T (Magnetom Skyra and Prisma, Siemens). The rods were made of titanium (Ti) with different alloys (Table 1): pure titanium alloy (TiCP), titanium aluminide (TiAl), cobalt-chromium (CoCr), and cobalt-chromium-molybdenum (CoCr Plus). The screw heads consisted of CoCr and the threads of Ti. The cross-links were also made from Ti.

**Table 1:** Implant characteristics

Type	Version	Manufacturer number	Length × diameter (mm)	Material
Rod	1553200500	0224584 W	Variable × 5.50	TiCP
	1554200500	0411249 W	Variable × 5.50	TiAl
	1555006150	0465970 W	Variable × 5.50	CoCr
	1556000500	0204211 W	Variable × 5.50	CoCr Plus
Screw	55840005540	H5344532	40.00 × 5.50	Head CoCr, thread Ti
Cross-link	8115528		44.00 × 17.00^1^	Ti

^1^length × width (mm)

The software SPSS Statistics version 25 (IBM) was used for statistical evaluation. A generalized linear model with post hoc Wald chi-squared tests was used to assess the influence of implant length, presence of cross-links, and magnetic field strength on RF-induced heating or susceptibility artifacts. The level of significance was set to *p* < 0.05. The study has been approved by the local ethics committee of the Jena University Hospital (registration number 2020-1849-Daten) and performed in accordance with its relevant guidelines and regulations. Written informed consent was waived by the committee, as it was a retrospective analysis of standard-of-care acquired image data.

### ASTM F2182: RF-induced heating

The RF-induced heating was measured according to ASTM F2182 [5] with a TrueFISP sequence at 1.5 T (TR/TE 3.04/1.52 ms, scan time 15 min) and 3 T (TR/TE 500/48 ms, scan time 15 min) with a whole-body specific absorption rate (SAR) of 2 W/kg for an assumed patient of weight 72.0 kg, height 166 cm, and age 40 years. The implant was hung from a wooden rod using thin plastic cords (Fig. 1e). This setup was immersed in a gel phantom (length 780 mm, width 560 mm, height 180 mm) filled with distilled water, 1.32 g/l sodium chloride (NaCl), and 10 g/l polyacrylic acid (PAA). The phantom was left in the scanner room for 24 h before measurements to adapt to the surrounding room temperature of around 23.5 °C. The temperature was measured every 2 s at four points (Fig. 1f) with fiberoptic sensors (Reflex Signal Conditioner, Neoptix). Twelve configurations were analyzed: two CoCr rods (see version 1555006150 in Table 1) without and with two cross-links (see version 8115528 in Table 1) in lengths of 430 mm with 30 screws, 400 mm with 28 screws, 300 mm with 20 screws, 260 mm with 16 screws, 200 mm with 12 screws, and 130 mm with 8 screws. For the configurations without cross-links, a piece of wood was placed between the two rods to serve as a placeholder, preventing contact between the two rods. The measurements (*n* = 3 for each configuration) were performed with the implants placed parallel to B_0_. In total, 72 (twelve configurations × three measurements per configuration × two B_0_) measurements were performed.

### ASTM F2119: susceptibility artifact

The susceptibility artifacts were measured according to ASTM F2119 [11] with a turbo spin echo sequence (TSE; TR 500 ms, TE 24–25 ms, echo train length 7) and gradient echo sequence (GRE; TR 100 ms, TE 15 ms, flip angle 30°). The voxel size was adjusted to the implant container size used for measurement (2 × 2 × 5 mm for large and 0.6 × 0.6 × 3 mm for small containers). The implant was hung from a wooden rod using thin plastic cords (Fig. 1e). This setup was immersed in a 1.5 g/l copper sulfate solution (CuSO_4_) in a large container (length 790 mm, width 390 mm, height 170 mm) for long implants (> 80 mm length) and in a small container (length 230 mm, width 160 mm, height 150 mm) for short implants (≤ 80 mm length). Including change of phase- and frequency-encoding direction, each measurement series consisted of 12 sequences (two pulse sequences, three slice directions, two frequency directions). The evaluation was performed with the tool MR-Susceptibility Artefact Measurement (SAM) [10] and histogram-based reference value. The artifact width is the maximum distance from the edge of the implant to the fringe of the resulting susceptibility artifact. In total, there were 18 configurations: without cross-links (one CoCr rod, see Table 1) and with two cross-links (two CoCr rods, see Table 1), each measured with lengths of 430 mm, 400 mm, 350 mm, 300 mm, 260 mm, 250 mm, 200 mm, 150 mm, and 130 mm without screws. The measurements were performed with the implants placed parallel to B_0_. Moreover, and according to the ASTM F2119, the susceptibility artifacts for all rods with a length of 80 mm (see Table 1) were evaluated without screws with the object placed parallel and perpendicular to B_0_ without and with metal artifact reduction technique “WARP” and a view-angle-tilting (VAT) of 30%. The screw and cross-link were measured with the object placed perpendicular to B_0_. In total, 768 MRI sequences were evaluated with approximately 3400 measurements.

### ASTM F2213 and F2052: magnetically induced torque and displacement force

The magnetically induced torque was measured according to ASTM F2213 [12] with a digital apparatus [18]. For this, an MRI-safe measuring platform was combined with a precision balance (PCB 1600-2, Kern & Sohn GmbH). The evaluation was performed for all rods with a length of 80 mm, the screw, and cross-link (see Table 1). Additionally, a CoCr rod with a length of 130 mm was examined to derive the magnetically induced torque for larger rod lengths. The torque was measured at 10-degree increments as the test object was rotated relative to B_0_ for a horizontal orientation. In total, 504 measurements were performed.

The magnetically induced displacement force was measured according to ASTM F2052 [17] with an MRI-safe holding structure that contained a protractor with 1° graduated markings. The deflection force was measured (*n* = 3) by the largest spatial gradient of B_0_ (11 T/m) at the entrance of the tube. The evaluation was performed for all rods with a length of 80 mm, the screw, and the cross-link (see Table 1). Additionally, a CoCr rod with a length of 130 mm was examined. In total, 42 measurements were performed.

## Results

The pedicle-screw-rod system could be reliably and safely examined in all combinations of length, configuration, and material in a B_0_ at 1.5 T and 3 T. There were no hazards or critical moments over the time.

### ASTM F2182: RF-induced heating

**Fig. 2:**
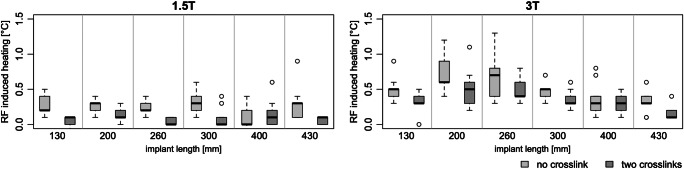
The maximum RF-induced heating of the titanium rods with CoCr alloy with no and two cross-links at 1.5 T (left) and 3 T (right) for different implant lengths. A spine model was used to bend the rods and position the screws anatomically correctly

The maximum RF-induced heating was 0.9 K at 1.5 T and 1.3 K at 3 T for all measurements (Fig. 2). On average, the RF-induced heating was 0.24 ± 0.08 K (without cross-links) and 0.09 ± 0.05 K (with cross-links) for 1.5 T and 0.50 ± 0.16 K (without cross-links) and 0.35 ± 0.14 K (with cross-links) for 3 T. RF-induced heating was significantly influenced by B_0_ (*p* < 0.001), implant length (*p* = 0.048), and presence of cross-links (*p* = 0.001). In general, the RF-induced heating was significantly greater without cross-links (1.5 T: 0.15 K, *p* < 0.001 with 95% CI [0.08; 0.22]; 3 T: 0.15 K, *p* < 0.01 with 95% CI [0.04; 0.27]). At a B_0_ of 1.5 T, no significant relationship between RF-induced heating and implant length was found (*p* values between 0.089 and 0.884). On the other hand, comparing the implant heating between different implant lengths at a B_0_ of 3 T, the RF-induced heating in implants of 200 mm was significantly greater than in longer (except 260 mm) or shorter implants (see Table 2).

**Table 2:** The RF-induced heating of titanium rods with CoCr alloy in different length at 3 T (compare with Fig. 2 right) were compared by a generalized linear model with post hoc Wald chi-squared tests. The level of significance was set to *p* < 0.05, and significant *p* values are shown in italics. At a B_0_ of 1.5 T, no significant relationship between RF-induced heating and implant length was found

Length (mm)	*p* value					
	130	200	260	300	400	430
130		*0.027*	0.064	1.000	0.338	0.059
200	*0.027*		0.461	*0.010*	*< 0.001*	*< 0.001*
260	0.064	0.461		*0.020*	*< 0.001*	*< 0.001*
300	1.000	*0.010*	*0.020*		0.160	*0.027*
400	0.338	*< 0.001*	*< 0.001*	0.160		0.112
430	0.059	*< 0.001*	*< 0.001*	*0.027*	0.112	

### ASTM F2119: susceptibility artifact

**Fig. 3:**
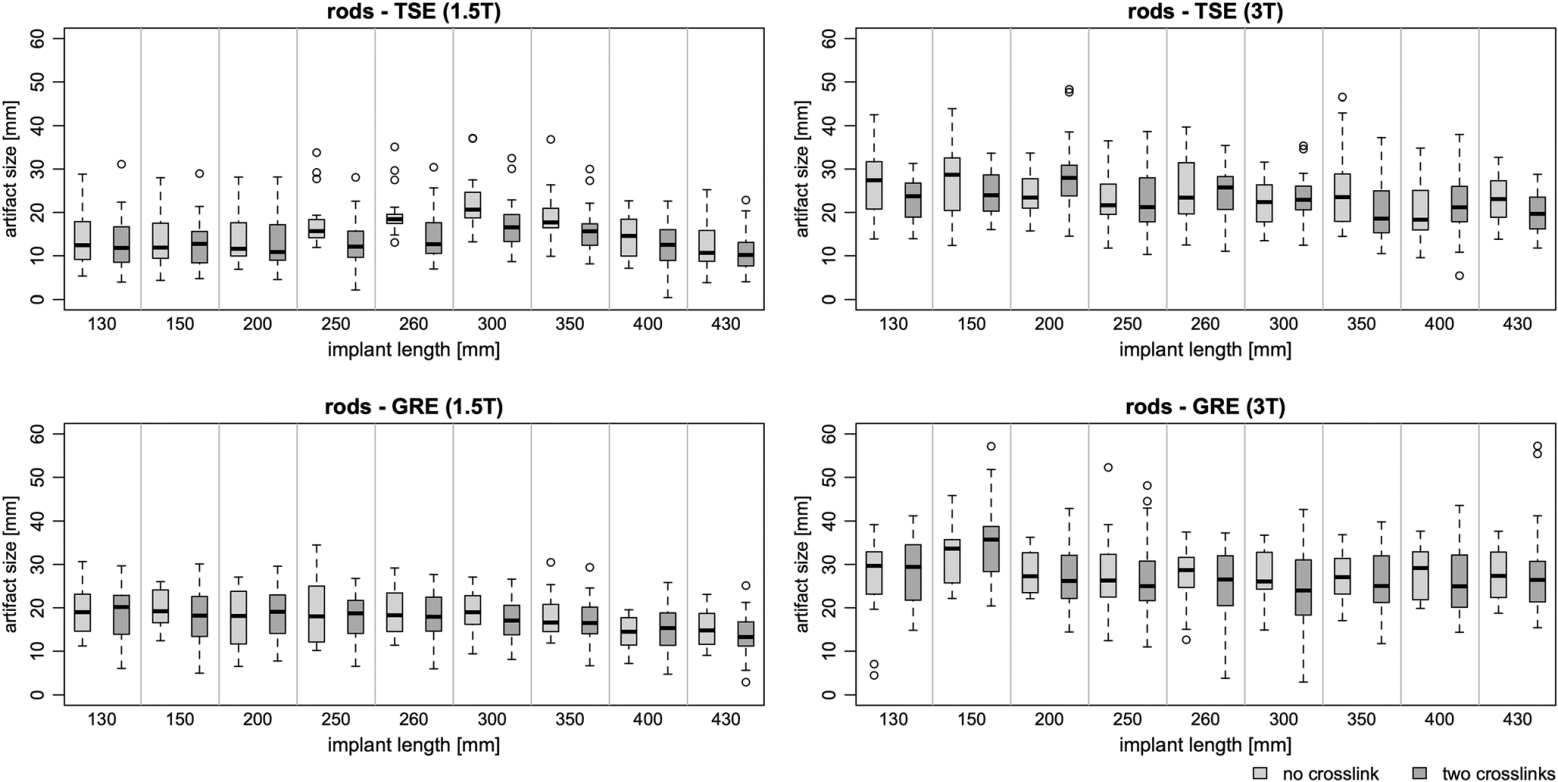
The maximum artifact width for a TSE (above) and a GRE (below) sequence for the titanium rods with CoCr alloy without and with two cross-links at 1.5 T (left) and 3 T (right) for different implant lengths. A spine model was used to bend the rods anatomically

The maximum artifact width (distance between implant surface and end of signal extinction) for different lengths of the anatomically bent Ti rods with CoCr alloy ranged between 14.77 ± 2.93 mm (TSE) and 17.49 ± 1.82 mm (GRE) for 1.5 T and between 23.67 ± 2.39 mm (TSE) and 27.77 ± 2.37 mm (GRE) for 3 T (Fig. 3). The largest artifact widths were measured at the edge of the implant. The maximum artifact widths were greater for GRE sequences than for TSE sequences (1.5 T: 1.7 mm, *p* < 0.001 with 95% CI [0.70; 2.80]; 3 T: 4.4 mm, *p* < 0.001 with 95% CI [2.90; 5.80]).

**Fig. 4:**
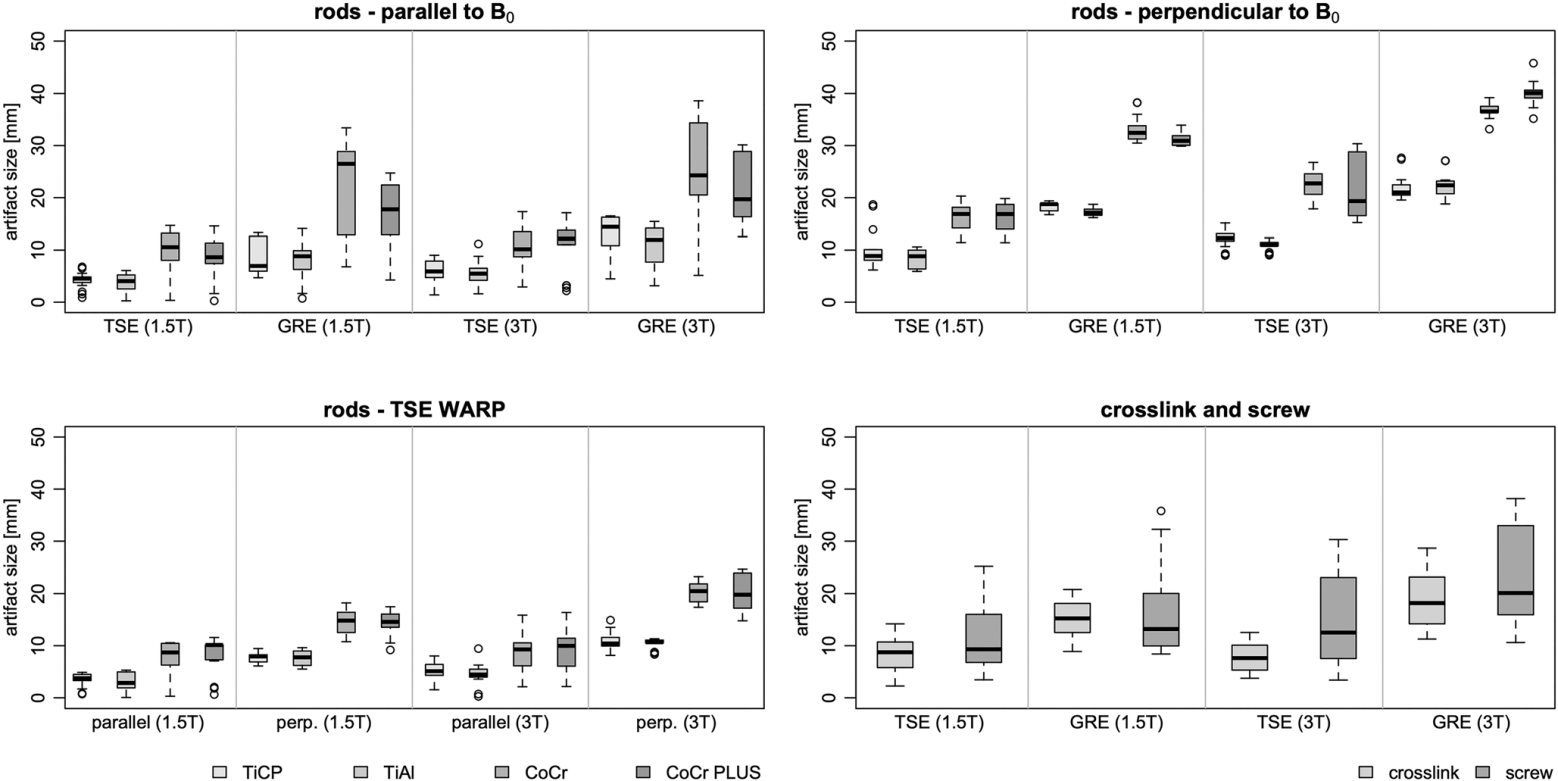
The maximum artifact width for a parallel (top left) and perpendicular (top right) alignment of the longitudinal axis to B_0_ for four straight rods with different alloys (see Table 1). Additionally, the metal artifact reduction technique WARP was evaluated (bottom left). At bottom right the maximum artifact width for the screw and cross-link (see Table 1) is shown

For the straight rods with different alloys, TiCP and TiAl showed the smallest and CoCr and CoCr Plus the largest artifact widths (Fig. 4, Table 1). With the objects placed parallel (perpendicular) to B_0_, the maximum artifact widths of the rods for a TSE sequence were as follows: TiCP, 4.97 ± 1.11 mm (11.01 ± 1.69 mm); TiAl, 4.67 ± 1.26 mm (9.58 ± 1.72 mm); CoCr, 9.76 ± 0.42 mm (19.42 ± 4.74 mm); and CoCr Plus, 9.66 ± 1.78 mm (19.01 ± 3.71 mm). For a GRE sequence, the maximum artifact widths were as follows: TiCP, 10.92 ± 3.53 mm (20.04 ± 2.49 mm); TiAl, 9.39 ± 1.66 mm (19.81 ± 3.67 mm); CoCr, 23.75 ± 1.51 mm (34.95 ± 2.61 mm); and CoCr Plus, 19.22 ± 3.19 mm (35.56 ± 6.27 mm). No significant difference in maximum artifact size was found between the materials TiCP and TiAl or between CoCr and CoCr Plus. However, the artifact size for group 1 (TiCP, TiAl) is significantly lower (9.84 mm, *p* < 0.001 with 95% CI [7.86; 11.81]) than for group 2 (CoCr, CoCr Plus).

Using the metal artifact reduction technique WARP with a VAT of 30%, the artifact size was reduced by 14 ± 6% (Fig. 4, bottom left). The application enables significant reduction of artifact widths (1.34 mm, *p* < 0.01 with 95% CI [0.34; 2.33]).

For the cross-link and screw, the artifact widths at 1.5 T (3 T) were 8.30 ± 0.61 mm (13.19 ± 2.54 mm) for a TSE and 17.05 ± 2.51 mm (19.85 ± 5.15 mm) for a GRE sequence (Fig. 4, bottom right).

### ASTM F2213 and F2052: magnetically induced torque and displacement force

For all investigated materials, the magnetically induced torque was less than 1 Nmm at both 1.5 T and 3 T. The maximum magnetically induced displacement angle at 1.5 T (3 T) was: TiCP, 1.0° ± 0.5° (2.0° ± 0.5°); TiAl, 1.5° ± 0.5° (2.0° ± 0.5°); CoCr, 2.0° ± 0.5° (6.0° ± 0.5°); and CoCr Plus, 2.0° ± 0.5° (6.0° ± 0.5°) for the rods with a length of 80 mm. For the rod with CoCr alloy and a length of 130 mm, the screw, and the cross-link, the maximum magnetically induced displacement angle at 1.5 T (3 T) was 2.0° ± 0.5° (7.0° ± 0.5°), 2.0° ± 0.5° (5.0° ± 0.5°), and 1.0° ± 0.5° (2.0° ± 0.5°), respectively.

## Discussion

The objective of this study was to evaluate the MRI safety and MR conditional status of a metallic pedicle-screw-rod system with different lengths (130 to 430 mm), configurations, and materials at magnetic field strengths of 1.5 T and 3 T based on standardized test methods of the ASTM. We found that even long implant constructs are MR conditional and safe in the standard clinical setting for MRI in patients with such implants with regard to RF-induced heating as well as magnetically induced torque and displacement force. However, we encountered clinically relevant susceptibility artifacts near the posterior spinal implants which were dependent on the field strength.

### ASTM F2182: RF-induced heating

In the present study, we examined metallic spinal implants ranging from 3 to 14 spinal segments (130–430 mm) in length, of the type usually employed for correction of scoliosis. Despite the considerable construct length and the large amount of metal, the maximum RF-induced temperature increase of the spinal implants in the worst case conditions (SAR 2 W/kg, 15 min MR acquisition) was only 1.3 K. The RF-induced heating was even less when two cross-links were used, although electrically closed loops (coils) can favor heating of the surroundings [19]. These results are in agreement with the findings of Tsukimura et al [1] for a 200-mm implant at 7 T. A review [20] demonstrated that low heating (up to 43 °C) for several hours causes no damage and that heating to 44 °C for 200 min leads only to transient erythema. Therefore, it can be assumed that adverse biological effects due to RF-induced heating of the investigated pedicle-screw-rod system in scoliosis patients, examined with a 1.5 to 3 T MRI in a supine position, become unlikely. In vitro studies [21, 22] on RF-induced heating of metallic orthopedic implants showed temperature increases of up to 9 or 14 K after MRI duration of only 15 min.

We found a critical length for effective heating [23–25] depending on the RF wavelength and implant length at 3 T. Nevertheless, the amount of heating is very small. A possible reason for this is that the distance between implant and RF source has a large influence on RF-induced heating. In general, the RF-induced heating are strongest near the walls and weakest in the center of the MRI apparatus [26]. When the patient is lying supine, the implant is located in the middle of the scanner and therefore has the lowest risk of heating. Tsukimura et al [1] did not find any significant difference between Ti rods with different alloys. Moreover, the Ti rods with CoCr alloy have the ability to produce higher correction rates in adolescent idiopathic scoliosis (AIS) and provide significant and stable spinal correction compared to Ti rods with TiCP alloy of the same diameter [27]. For these reasons, this study carried out the complex measurement only for Ti rods with CoCr alloy in different configurations.

The automatic SAR limitation of the MR system [28] is country-specific designed for a maximum temperature (e.g., 24 °C) and a maximum relative humidity (e.g., 60%) in the magnetic room. If the room temperature and/or the humidity is higher than specified above, compliance with the SAR limit values according to the International Electrotechnical Commission (IEC) or Federal Food and Drug Administration (FDA) guideline may no longer be ensured and therefore the SAR limits are reduced accordingly. This has the consequence that maybe some MR sequences are not available anymore. Only the sequence adaptation for SAR reduction remains possible. Besides this, the present and numerous other studies publications [1, 2, 7, 21–23, 26, 29, 30] were based on the original ASTM standard F2182 which suggests a whole-body SAR of approximately 2 W/kg.

### ASTM F2119: susceptibility artifact

Susceptibility artifact can strongly reduce the diagnostic value of MRI, especially if the region of interest is close to the implant. This is the case, for example, for patients in whom postoperative hematoma or recurrent spinal tumor is suspected. Therefore, it is important to know which parameters influence the artifacts, in order to minimize it. In general, the size of susceptibility artifacts depends on the chemical elements present in the construct (implant material composition) [31], the geometry and volume of the implant [32], the magnetic field strength [33], the orientation of the implant in relation to B_0_ [31], the MRI sequence used [32], and the sequence parameters [31, 33, 34]. Comparison of the different rods in this study indicated that ferromagnetic cobalt is particularly likely to increase the susceptibility artifact. Avoiding the use of cobalt alloys might therefore reduce artifact size in cases where regular evaluation of the region adjacent to the implant is necessary.

Apart from the implant material, the size of susceptibility artifacts depends on the orientation of the implant in relation to B_0_, in agreement with previous studies [9, 31]. The susceptibility artifacts will be lowest if the longest axes of the rod, screw, and cross-link are parallel to B_0_. Such alignment is not possible for an anatomically bent pedicle-screw-rod system. The bent section is no longer parallel to B_0_ and the angle can be of different sizes. Therefore, the rods were measured parallel (best case) and perpendicular (worst case) to B_0_ as recommended by the standard ASTM F2119 [11]. In spinal implants the screws and cross-links can almost always only be positioned perpendicular to B_0_, so for these components only this orientation was examined.

**Fig. 5:**
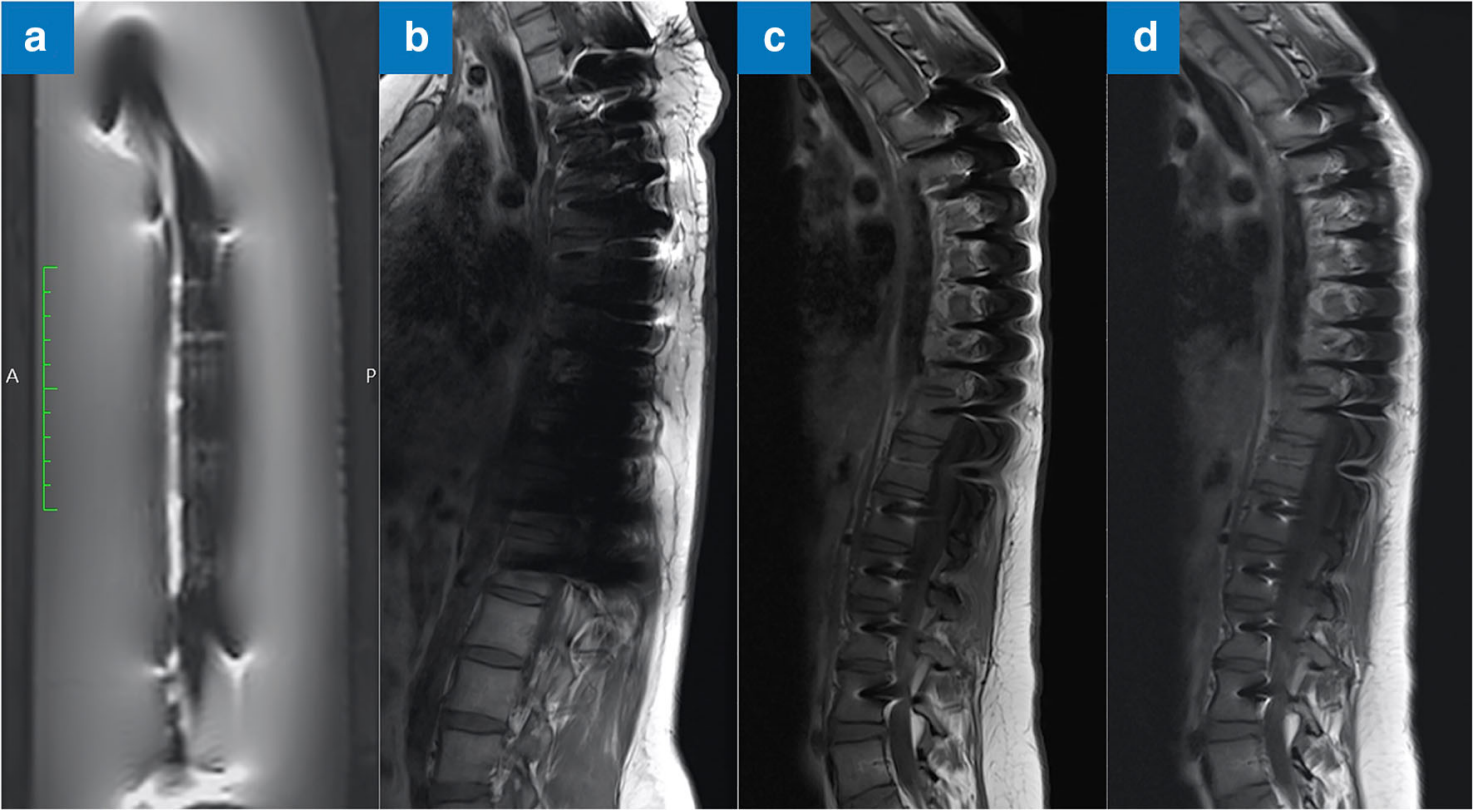
MR images of a pedicle-screw-rod system approximately 300 mm long in a phantom (**a**, without screws) and in two volunteer patients at 3 T (**b**) and 1.5 T (**c**). Additionally, the effect of the artifact reduction method WARP is shown for 1.5 T (**d**)

Magnetic field strength and sequence parameters also had a major effect on the susceptibility artifacts in the present study, indicating that lower (versus higher) field strength and TSE (versus GRE) sequences are beneficial with regard to artifact size. In Fig. 5, it is shown that especially at 3 T (Fig. 5a and b) the diagnostic value of MRI is strongly reduced in the region near the spinal implant. However, WARP can help reduce the artifacts caused by metal implants and therefore allows improved soft tissue evaluation [35, 36]. Nevertheless, for spinal implants, WARP does not automatically enable diagnostic evaluation of adjacent tissues (Fig. 5d). WARP can be used in three methods [28]: WARP with high bandwidth to reduce geometric distortions and changes in contrast, as well as, WARP with VAT technology to correct geometric in-plane distortions and WARP with VAT and slice encoding for metal artifact correction (SEMAC) to correct geometric through-plane distortions. The outcome of WARP with VAT depends largely on the type and orientation of the implant as well as other imaging parameters. Additionally, for warp with VAT and SEMAC, the number of SEMAC phase-encoding steps required depends on the size, shape, and material of the implant and can vary from patient to patient. Optimization of the WARP algorithm might lead to much better results than shown here but did not form part of the study. For departments where MRI is routinely performed in patients with scoliosis, we urgently suggest implementation of such routines.

### ASTM F2213 and F2052: magnetically induced torque and displacement force

Under the conditions of the present study, the magnetically induced torque was negligible. In general, magnetically induced torque is minimal if the longitudinal axis of the test object is parallel to B_0_. This is almost exclusively the case for the rods (with the patient supine). Additionally, there is a linear relationship between the length of the test object and the magnetically induced torque [18]. However, even with vertical alignment of a rod 130 mm long, the magnetically induced torque was below 1 Nmm.

Regarding the magnetically induced displacement force, we found significantly greater angles for 3 T than for 1.5 T. These results are basically in agreement with the findings of Tsukimura et al [1] at 3 T. Nevertheless, since the angles for all test objects were far below 45°, they can be classified as safe according to the ASTM [17].

## Limitations

The present study is not without limitations. First, all measurements were performed in a phantom. The real-life situation in patients can differ from idealized protocols. However, well-accepted standard protocols were applied (ASTM). Second, all of the spinal implants tested were manufactured by the same company. Other companies may use different alloys for their implants, and this could influence the results, especially if the content of cobalt or other ferromagnetic metals is higher. Furthermore, we performed no optimization of the WARP sequences. Further studies are required to investigate the benefits of this technology for pedicle-screw-rod constructs.

## Conclusion

In summary, large spinal implants are not necessarily a contraindication for MRI. Although susceptibility artifacts can severely limit the diagnostic capacity of MRI near the implants, the examination of other regions is safely possible. The RF-induced heating of the implant and the magnetically induced torque and displacement force are all at acceptable levels with the patient in the supine position. According to ASTM F2503 [37], the pedicle-screw-rod system is MR conditional in the following setting: patient in supine position, B_0_ of 1.5 T or 3 T, and length of TiCP, TiAl, CoCr, or CoCr Plus rods between 130 and 430 mm.

## Abbreviations

ASTM: American Society for Testing and Materials
CoCr Plus: Cobalt-chromium-molybdenum
CoCr: Cobalt-chromium
RF: Radio frequencies
Ti: Titanium
TiAl: Titanium aluminide
TiCP: Pure titanium alloy
